# Investigating clinical heterogeneity in systematic reviews: a methodologic review of guidance in the literature

**DOI:** 10.1186/1471-2288-12-111

**Published:** 2012-07-30

**Authors:** Joel J Gagnier, David Moher, Heather Boon, Joseph Beyene, Claire Bombardier

**Affiliations:** 1 Departments of Orthopaedic Surgery and Epidemiology, University of Michigan, 24 Frank Lloyd Wright Drive, Ann Arbor, MI, USA; 2 Clinical Epidemiology Program, Ottawa Health Research Institute, Ottawa, ON, Canada; 3 Department of Epidemiology & Community Medicine, Faculty of Medicine, University of Ottawa, Ottawa, ON, Canada; 4 Leslie Dan Faculty of Pharmacy, University of Toronto, Toronto, ON, Canada; 5 Child Health and Evaluative Sciences, The Hospital for Sick Children, Toronto, ON, Canada; 6 Health Policy, Management & Evaluation, Faculty of Medicine, University of Toronto, Toronto, ON, Canada

## Abstract

**Background:**

While there is some consensus on methods for investigating statistical and methodological heterogeneity, little attention has been paid to clinical aspects of heterogeneity. The objective of this study is to summarize and collate suggested methods for investigating clinical heterogeneity in systematic reviews.

**Methods:**

We searched databases (Medline, EMBASE, CINAHL, Cochrane Library, and CONSORT, to December 2010) and reference lists and contacted experts to identify resources providing suggestions for investigating clinical heterogeneity between controlled clinical trials included in systematic reviews. We extracted recommendations, assessed resources for risk of bias, and collated the recommendations.

**Results:**

One hundred and one resources were collected, including narrative reviews, methodological reviews, statistical methods papers, and textbooks. These resources generally had a low risk of bias, but there was minimal consensus among them. Resources suggested that planned investigations of clinical heterogeneity should be made explicit in the protocol of the review; clinical experts should be included on the review team; a set of clinical covariates should be chosen considering variables from the participant level, intervention level, outcome level, research setting, or others unique to the research question; covariates should have a clear scientific rationale; there should be a sufficient number of trials per covariate; and results of any such investigations should be interpreted with caution.

**Conclusions:**

Though the consensus was minimal, there were many recommendations in the literature for investigating clinical heterogeneity in systematic reviews. Formal recommendations for investigating clinical heterogeneity in systematic reviews of controlled trials are required.

## Background

Systematic reviews sometimes apply statistical techniques to combine data from multiple studies resulting in a meta-analysis. Meta-analyses result in a point estimate, the summary treatment effect, together with a measure of the precision of results (e.g., a 95% confidence interval). These measures of precision represent the degree of variability or heterogeneity in the results among included studies. There are several possible sources of variability or heterogeneity among studies that are included in meta-analyses. Variability in the participants, the types or timing of outcome measurements, and intervention characteristics may be termed clinical heterogeneity; variability in the trial design and quality is typically termed methodological heterogeneity; variability in summary treatment effects between trials is termed statistical heterogeneity [1]. Methodological and clinical sources of heterogeneity contribute to the magnitude and presence of statistical heterogeneity [1].

Methodological heterogeneity hinges on aspects of implementation of the individual trials and how they differ from each other. For example, trials that do not adequately conceal allocation to treatment groups may result in overestimates in the meta-analytic treatment effects [2]. Significant statistical heterogeneity arising from methodological heterogeneity suggests that the studies are not all estimating the same effects due to different degrees of bias.

Clinical heterogeneity arises from differences in participant characteristics (e.g., sex, age, baseline disease severity, ethnicity, comorbidities), types or timing of outcome measurements, and intervention characteristics (e.g., dose and frequency of dose [1]). This heterogeneity can cause significant statistical heterogeneity, inaccurate summary effects and associated conclusions, misleading decision makers and others. As such, systematic reviewers need to consider how best to handle sources of heterogeneity [1]. For example, preplanned subgroup analyses, stratifying for similar characteristics of the intervention and participants, could tease-out important scientific and clinically relevant information [3].

Systematic reviews are frequently recognized as the best available evidence for decisions about health-care management and policy [3-7]. Results of systematic reviews are often incorporated into clinical practice guidelines [5] and required in funding applications by granting agencies [6]. In spite of all this it appears health-care professionals and policy makers infrequently use systematic reviews to guide decision-making [8].

A limitation of many systematic reviews is that their content and format are frequently not useful to decision makers [8]. For example, while some guidance exists describing what to include in reports of systematic reviews (e.g., the PRISMA statement [9]), characteristics of the intervention that are necessary to apply their findings are infrequently provided [10-13]. This has led to some preliminary work on how to extract clinically relevant information from systematic reviews [14]. Furthermore, systematic reviews commonly show substantial heterogeneity in estimated effects (statistical heterogeneity), possibly due to methodological, clinical or unknown features in the included trials [15]. While guidance exists on the assessment and investigation of methodological [1] and statistical heterogeneity [1,16], little attention has been given to clinical heterogeneity.

We report a systematic review of suggested methods for investigating clinical heterogeneity in systematic reviews of controlled clinical trials. We also provide some guidance for systematic reviewers.

## Methods

This project identified resources giving recommendations for investigating clinical heterogeneity in systematic reviews. We extracted their recommendations, assessed their risk for bias, and categorized and described the suggestions.

### Search

The following databases were searched: Medline (to October 29, 2010), EMBASE (to Oct 30, 2010), CINAHL (1981 to Oct 30, 2010), Health Technology Assessment (to Oct 29, 2010), the Cochrane Methodology Register (to Oct 29, 2010), and the CONSORT database of methodological papers (to October 30, 2010). A library and information scientist was consulted to create sensitive and specific searches, combining appropriate terms and extracting new terms from relevant studies for each database. The following search terms were used in the various databases and at various stages of the search: heterogeneity, applicability, clinical, assessment, checklist, guideline(s), scale, and criteria. The “adjacent” or “within X words” tools were used for the terms “clinical” and “heterogeneity” for all databases. In addition, we used the PubMed related-links option that identifies indexed studies on similar topics or having similar indexing terms to include a broad range of papers that might be indirectly related to clinical heterogeneity. Appendix A contains details of the electronic searching. One investigator (JG) contacted representatives of the Cochrane Collaboration, the Campbell Collaboration, the Agency for Healthcare Research and Quality (AHRQ) and a selection of experts identified through an initial review of the literature to suggest relevant articles, guidelines, position papers, textbooks or other experts in the area. We also reviewed the *Cochrane Handbook*, the Campbell Collaboration methods guides, and the AHRQ comparative effectiveness section for any guidance on clinical heterogeneity and searched reference lists of all retrieved resources.

The overall process consisted of a “snowballing” technique of seeking information on the topic, by which we asked experts to refer us to other experts or resources, and so on, until each new resource yielded a negligible return. Several individuals with expertise in the area of systematic reviews (JG, DM, JB, CB) met to identify key textbooks to include out of thier personal knowledge of textbooks in the area. These individuals presented what each felt were key textbooks in the area and then debated the merits of each, finally coming to a consensus-based decision on which to include. In general, these methods allowed us to include a broad array of resources related to investigating clinical heterogeneity in systematic reviews.

### Inclusion criteria

Clinical heterogeneity is defined as differences in participant, treatment, or outcome characteristics or research setting. We included any methodological study, systematic review, guideline, textbook, handbook, checklist, scale, or other published guidance document with a focus on assessing, measuring, or generally investigating clinical heterogeneity between or within controlled clinical trials included in systematic reviews. This included quantitative, qualitative, graphical or tabular techniques, suggestions or methods.

### Exclusion criteria

Systematic reviews of interventions for efficacy were excluded.

### Data extraction

A data extraction form was developed and piloted independently by two individuals (JG, DM) on a random selection of 10 included resources. Extractions were checked for consensus and the form revised according to the feedback provided. One person extracted information for all included studies (JG) regarding why the authors sought to assess clinical heterogeneity; what “criteria” were used to assess clinical heterogeneity; how these were developed; the definition of clinical heterogeneity used by the authors; any graphical, tabular or other display/summary methods; statistical recommendations; reported methods used; empirical validation performed on the “criteria”; examples of implementing the methods; and recommendations on how the assessments are to be used in systematic reviews. All extractions were checked for accuracy by another individual (DM).

### Synthesis methods

We thematically grouped the retrieved resources, suggestions or techniques (e.g., statistical versus qualitative recommendations), described the recommendations, highlighted any empirical support cited for each recommendation, and made an overall summary of the recommendations.

### Assessment of method validation

Four individuals (JG, DM, JB, CB) met several times to discuss how to rate the variety of resources retrieved. These individuals came to a consensus that there were several classes of resources that did not have any accepted risk-of-bias assessment tools or instruments (e.g., textbooks, narrative reviews, learning guides, expert opinions). Therefore instead, of assessing “risk of bias” of these articles, we chose to determine if specific methods have been validated. Resources were considered validated if they had a clear rationale or reported empirical evidence for that recommendation (e.g., reference to previous empirical work or a test of the method with empirical or simulated data). One individual (JG) assessed the method of validation of each of these included resources.

## Results

**Figure 1:**
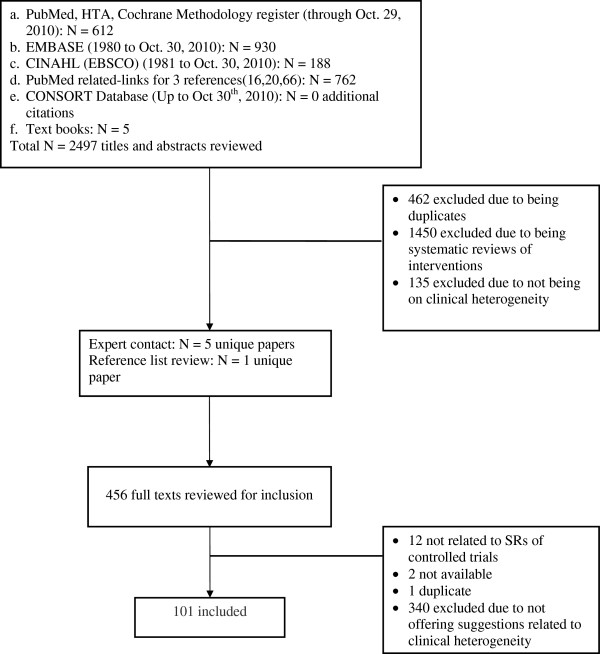
Search and inclusion results.

Our searches identified 2497 unique titles and abstracts; after screening, 101 papers were included in the review [17-117]. These resources included statistical papers, methodological reviews, narrative reviews, expert opinions, learning guides, consensus-based guidelines and textbooks. Figure 1 describes details of the search and screening results. The very few disagreements on inclusion were easily resolved through discussion. Sixty-four (64.6%) of the resources (statistical, methodological, consensus guideline resources) were assessed for validation. Forty-one (64.1%) of these references were evaluated as being sufficiently validated.

Table 1 describes some basic characteristics of the included resources. The most common type of resource was statistical papers (42.4%), with narrative reviews/expert opinion papers being the next most common (29.3%). Most of the papers were published in the 2000s (70.1%), and statistical methods for investigating clinical heterogeneity were the most frequent types of suggestions across resources (73.7%). Table 2 reports a list of clinical variables suggested for investigating clinical heterogeneity and the number and types of resources suggesting each. General suggestions of clinically related variables, without identification of specific clinical covariates, were the most common across all included resources. Most suggestions were within distinct categories: participant level (e.g., age), intervention level (e.g., dose), or outcome level (e.g., event type, length of follow-up) covariates. A number of resources (N = 14) reported control event rate/baseline risk as being a covariate worth investigating.

**Table 1:** Descriptive characteristics of included resources that reported recommendations for investigating clinical heterogeneity in systematic reviews of controlled clinical trials (N = 101)

**Descriptive Characteristics**		**N**^**1**^
**Type of publication**	Statistical paper	44
	Narrative review or expert opinion	29
	Methodological review	14
	Consensus-based guideline	9
	Textbook	5
**Decade of publication**	2000s	70
	1990s	27
	1980s	4
**Guidance on statistical methods**		75
**Clinical variables (general**^**2**^**or specific) recommended**		39
**Process for choosing clinical variables recommended**		28

1. The number (N) of resources equals the percentage of resources since we include 101 total resources.

2. The term “general” means that the resource listed the term “patient”, “intervention”, or “outcome” as a category from which to consider covariates without suggesting specific variables.

**Table 2:** Types of clinical covariates suggested across all resources

**General Category**	**Specific Covariate**	**Number of Resources Recommending**^**1**^
Patient level	General^2^	15
	Age	7
	Baseline severity	3
	Sex/gender	4
	Ethnicity	2
	Comorbidities	2
	Other disease features	2
Intervention level	General	13
	Dose	8
	Duration	5
	Brand	3
	Co-interventions	3
	Intensity	3
	Timing	3
	Route	2
	Compliance	2
	Others unique to the intervention	2
	Frequency	1
	Comparator/control	1
Outcome level	General	6
	Event type	5
	Length of follow-up	4
	Outcome measure type	3
	Outcome definition	3
	Timing	2
	Repeated outcome measurements	1
Control event rate / baseline risk		14
Research setting		4
Comparison conditions		3
Early stopping rules		1
Population risk		1

1. The number (N) of resources equals the percentage of resources since we include 101 total resources.

2. The term “general” means that the resource listed the term “patient”, “intervention”, or “outcome” as a category from which to consider covariates without suggesting specific variables.

Table 3 lists recommendations regarding the process of choosing clinical characteristics to investigate. Five or more resources suggested the following: *a priori* choice of clinical covariates (e.g., in the review protocol); look at forest plots for trials that may contribute to heterogeneity and then look for clinical characteristics therein; proceed with investigation regardless of results of formal testing for statistical heterogeneity; base clinical covariates on a clear scientific rationale (e.g., a pathophysiological argument); investigate a small number of covariates; base each covariate suggestion on an adequate number of trials (e.g., 10 trials was a common suggestion); use caution when interpreting the findings of investigations; consider the results of such investigations as exploratory, hypothesis generating and observational; and consider confounding between covariates.

**Table 3:** Recommendations regarding the methods of choosing or identifying clinical covariates for investigation and interpretation of the findings

**General Category of Recommendation**	**Specific Recommendation**	**Number of Resources**^**1**^	**Citations**
**When to identify covariates in the review process**	A priori (e.g., in protocol)	17	76, 92,93,95, 100, 98, 18, 26, 39, 40, 30, 59, 29, 31, 46, 94, 114
**How to find important clinical covariates from trial information**	Looking at forest plots (variation in point estimates/CI overlap/ adding a vertical line for levels of some clinical variable)	6	92, 98, 93, 97, 98, 94
	Proceed regardless of formal testing of statistical heterogeneity	5	35, 92, 97, 98, 29
	Looking at L’Abbe plots	4	98, 45, 93, 98
	Influence plot	3	98, 54, 85
	Looking at summary tables	2	92, 24
	Looking at funnel plots	2	49, 98
	Use conceptual frameworks to facilitate choice of covariates (i.e., using taxonomies for active ingredients)	2	98, 112
	I^2^ (look at the change in statistical heterogeneity by adding subgroups)	2	87, 100
	Plot of effect size against individual covariates	1	48
	Using an adaptation of multidimensional scaling (CoPlot)	1	55
	Plot of normalized z-scores	1	93
	Radial/Galbraith plot	1	93
	Frequency distributions	1	98
	Dose-response graph	1	3?
	Use P.I.C.O. model to guide choice of characteristics	1	115
	Use causal mediating processes	1	113
	Treat strata within trials as separate studies; these subgroups if similar across studies can be combined	1	46
**Rationale for choice of covariate**	Scientific (e.g., pathophysiological, pharmacologic argument)	10	7,76,92,93, 100, 18, 26, 59, 31, 115
	Previous research (e.g., large RCT)	3	76, 68, 100
	Clinical grounds	2	96, 100
	Indirect evidence	1	59
**Personnel**	Use of clinical experts	2	21, 115
	Blind to results of trials	1	35
**Number of covariates/trials needed**	Small number of covariates	7	92, 95, 100, 18, 26, 31, 94
	Each covariate investigation should be based on an adequate number of studies (e.g., 10 for every moderator)	6	100, 59, 50, 94, 115
	Investigators must report actual number of covariates investigated for reader to determine the potential for false-positives	1	115
**Number of outcomes to investigate**	Restrict investigations to small number of outcomes (e.g., primary)	1	26
	Limit to central question in the analysis	1	94
**Interpretation of results of investigations**	Use caution (4 resources note especially with post hoc testing)	12	100, 18, 29, 31, 85, 16, 20, 23, 25, 61, 32, 35
	Observational only	6	59, 23, 94, 98, 100, 114
	Exploratory or hypothesis generating only	4	32, 100, 40, 94
	Consider confounding between covariates	4	100, 50, 115, 59
	Consider artifactual causes of between-study variation	2	6, 98
	Consider biases (e.g., misclassification, dilution, selection)	2	93, 115
	Look at magnitude of the effect and the 95% CI; not just effect and p-value; consider precision of the subgroup effects (e.g., sample sizes in the studies dictate precision of the subgroup effects)	2	100, 115
	Seek evidence to justify claims of subgroup findings	1	26
	Identify knowledge gaps in the investigations	1	24
	Consider effect of variability within studies	1	19
	Consider if the magnitude is clinically important (i.e., differences in effect between subgroups)	1	100
	Think through causal relationships, especially directionality	1	113
	Use caution with variables grouped after randomization	1	23
	Consider parabolic relationships (i.e., beyond linear regression)	1	115
	Be cautious not to say there is a consistency of effect if no subgroup effects are found	1	115
**Descriptive methods**	Perform a narrative synthesis of these investigations	4	115, 98, 27, 100
	Other: 1. idea webbing, 2. qualitative case descriptions, 3. investigator/methodological/conceptual triangulation	1	98
**Use of types of data**	Aggregate patient data for trial level covariates	4	23, 25, 118, 46
	Only group characteristics derived prior to randomization (e.g., stratifying)	2	23, 46
	Individual patient data for participant level covariates	1	59
	Individual patient data only for all covariates where possible	1	59

1. The number (N) of resources equals the percentage of resources since we include 101 total resources.

Table 4 summarizes the types of statistical methods suggested for investigating clinical heterogeneity characteristics and the number of resources suggesting each. Many included resources made some mention of statistical methods of investigating aspects of clinical heterogeneity (N = 69/99, 69.7%). Also, many of these resources made general suggestions regarding the use of subgroup analyses (N = 18) and meta-regression (N = 16); however, the majority of these did not offer any specific recommendations. A wide variety of meta-regression techniques were suggested, many of which included simulated evidence or other forms of empirical testing. Several Bayesian approaches were suggested as well as several methods for individual patient data analysis [34,48,58,63,66,69,71,75,95]. Four textbooks appeared to be relatively comprehensive in their treatment of statistical recommendations [93-95,114].

**Table 4:** Statistical suggestions for investigating aspects of clinical heterogeneity

**General Category of Statistical Method**	**Specific Method Suggested**	**Number of Resources**^**1**^	**Citations**
**Subgroup analyses**	General	18	60, 2324, 25,46, 48, 50, 75, 92, 94, 93, 27, 97, 100, 98, 115, 105, 19
	Hierarchical testing procedure based on the heterogeneity statistic Q	1	114
	Combining subgroups across studies (i.e., in stratified studies)	1	114
**Moderator Analyses**			
1. ANOVA^2^ analogue (e.g., a categorical moderator)		4	48, 94, 95, 114
2. Meta-regression	General mention	16	19, 60, 6, 24, 2528, 31,32,43, 50, 75, 94, 95, 100, 98, 93, 1325, 418
	Fixed effects model (general)	4	92, 93, 94, 95
	Bayesian models (general)	4	66, 71, 124, 95
	New maximum likelihood method	2	60, 124
	New weighted least squares model	2	58, 67
	Random effects model (general)	2	67, 114
	Random effects model for IPD^3^	2	58, 61
	Permutation-based resampling	2	31, 43
	Other nonparametric (e.g., fractional polynomials, splines)	2	69, 85
	Mixed effects model	2	38, 114
	New variance estimators (for covariates)	2	77, 84
	Methods for measurement of residual errors	2	59, 41
	Bayesian model in the presence of missing study-level covariate data	1	110
	Semi-parametric modeling (general)	1	80
	Fixed effects generalized least squares model	1	68
	Hierarchical regression models	3	60, 64, 124
	Random effects model with new variance estimator	1	70
	Logistic regression with binary outcomes	1	25
	Interaction term for meta-regression model	1	95
	Consider nonlinear relationships (e.g., use quadratic or log transformations)	1	48
	Bayesian model for use in meta-analyses of multiple treatment comparisons	1	111
3. Multivariate analyses		1	48
4. Multiple univariate analyses with Bonferroni adjustments		1	48
5. Meta-analysis of interaction estimates		1	61
6. Model to include the repeated observations (time as a variable) using IPD		1	109
7. Z test		1	125
**Bayesian Approaches**			
1. Hierarchical Bayesian modeling		2	44, 48
2. Random effects models		1	63
**Data Specific Approaches**			
1. IPD analyses	General	5	75, 76, 95, 97, 23
	Regression	1	61, 46
	Adding a treatment-covariate interaction term	1	95
2. Combination of IPD & APD^4^	Two-step models	2	74, 78
	Multi-level model	2	69, 100
	Meta-analysis of interaction estimates	1	61
**Other Approaches**			
Models for control event rate / baseline risk	General (e.g., control event rate)	10	63, 24, 71, 81, 79, 93, 100, 19, 78, 111
Structural equation modeling (SEM)	Integration of SEM with fixed, random and mixed effects meta-analyses	1	42
Mixed treatment comparisons combined with meta-regression		1	72
Combining regression coefficients from separate studies		1	64

1. The number (N) of resources equals the percentage of resources since we include 101 total resources; 2. ANOVA = analysis of variance; 3. IPD = individual patient data; 4. APD = aggregate patient data.

Overall, we felt that there was some consensus across the resources regarding planning investigations, the use of clinical expertise, the rationale for choice of covariate, how to think through types of covariates, making a covariate hierarchy, post hoc covariate identification, statistical methods, data sources and interpretation of findings (See Table 5). We summarize the common recommendations that appeared in the literature to offer some preliminary guidance for systematic reviewers in Table 5 and we elaborate on several key areas in the discussion section below.

**Table 5:** Summary of recommendations for investigating clinical heterogeneity in systematic reviews

**Recommendation Category**	**Recommendation Description**
A-priori planning	1. All plans for investigating clinical heterogeneity should be made explicit, a-priori (e.g., in the protocol for the systematic review).
Clinical expertise	2. The review/investigative team should include clinical experts or state a plan for consulting clinical experts during the review protocol development and implementation (e.g., when choosing clinical covariates and when interpreting the findings).
Covariate rationale	3. Clinical covariates should be chosen that have a clearly stated rationale for their importance (e.g., a pathophysiological argument, reference to the results of a previous trial).
Thinking through covariate categories	4. Review teams should think through the following categories to determine if related covariates might logically influence the treatment effect in their particular review: participant level, intervention level, outcome level, research setting, or others unique to their research question.
Covariate hierarchy	5. A logical hierarchy of clinical covariates should be formed and investigated only if there is sufficient rationale and a sufficient number of trials available (10 trials per covariate).
Post hoc covariate identification	6. State any plans to include additional covariates after looking at the data (post hoc) from included studies (e.g., forest plots, radial plots) and how they plan to do this.
Statistical methods	7. Describe a-priori the statistical methods proposed to investigate identified covariates. Refer to accepted texts or published papers in the area to be sure to implement these methods in a valid manner. Include an individual with experience in conducting these analyses.^1^
Data sources	8. Aggregate patient data: Reasonable for investigating trial level covariates
	9. Individual patient data: Consider when investigating participant level covariates (otherwise results are subject to ecologic bias)
Interpretation	10. A. Protocol: Describe how the results of any findings are going to be interpreted and used in the overall synthesis of evidence. B. Review: Consider the observational nature of these investigations; consider confounds and important potential biases; consider magnitude of the effect, confidence intervals and directionality of the effect.

^1^ We do not provide detailed recommendations for statistical analyses here because of the breath and complexity of this topic. Instead we suggest that one refer to accepted resources and well-trained individuals with expertise in the area.

Sources appearing to be the most comprehensive in their discussion of recommendations for investigating clinical heterogeneity included the *Cochrane Handbook*[100] and the Centre for Reviews and Dissemination’s *Guidance For Undertaking Reviews In Health Care*[98] and the AHRQ *Comparative effectiveness review methods: clinical heterogeneity*[115].

## Discussion

A variety of decisions must be made when performing a systematic review. One such decision is how to deal with obvious differences among and within trials. Though a significant test for the presence of statistical heterogeneity (e.g., Q test) and a large degree of heterogeneity (e.g., I^2^ > 75%) might obligate a reviewer to look for covariates to explain this variability, a nonsignificant test or a small I^2^ (e.g., <25%) does not preclude the need to investigate covariate treatment effect interactions [35,92,97,100]. That is, even with low statistical heterogeneity, there may still be factors that influence the size of the treatment effect, especially if there is a strong argument (i.e., pathophysiologic or otherwise) that some variable likely does have such an influence.

Observed or expected heterogeneity of treatment effects can be handled in several ways. The heterogeneity can be ignored and a meta-analysis conducted with a fixed-effects or random-effects model, or one can attempt to explain the heterogeneity through subgroup analyses, meta-regression or other techniques [25]. The latter moves the review away from overall statements of evidence to increasingly clinically applicable results and conclusions as well as new hypotheses for future research [75]. [Anello and Fleiss 28] make a clear distinction between meta-analyses with a goal of arriving at a common summary estimate of effect (“analytic meta-analyses”) and those focused on explaining why the effect sizes vary (“exploratory or causal meta-analyses”). The choice between these depends on the objective of the review, but it is clear that meta-analyses are more applicable to decision making (e.g., clinical, policy) when they are exploratory in nature [14,28,53,75,99]. The trials included in a systematic review may be so very similar that the summary effect estimate is the most reasonable and applicable metric [114]. But these cases are very rare, and therefore we would expect most questions asked and tested through meta-analytic methods should concern possible reasons for variation in effect [114].

Many resources were found that suggested methods for carrying out investigations of clinical heterogeneity in systematic reviews [17-102]. There was great variety in the types of resources identified (statistical papers to commentaries) and in their potential for risk of bias. It was decided early to include any resource, no matter the design, methods, or publication type. For this reason many of the included resources might normally be considered at a high risk of bias (e.g., narrative reviews, expert opinions, learning guides and commentaries) and thus providing suggestions of questionable validity. But it was felt that these types of resources might provide the most valuable information on the subject of clinical heterogeneity. That is, investigating, and in particular choosing which clinical characteristics to investigate, requires clinical expertise, or at a minimum, knowledge of empirical evidence of some covariate of importance. The inclusion of these resources could be viewed as a drawback, but we saw it as a strength of this research. It was these resources that provided most of the suggestions regarding the methods for choosing or identifying clinical covariates to investigate (Table 3). The consensus-based guidelines provided most of the suggestions regarding the process of choosing or identifying clinical covariates, and the statistical papers, as might be expected, covered the majority of the specific statistical suggestions; but the textbooks also offered many suggestions in both areas. There was some consensus across resources, but only a small number of resources included a relatively comprehensive set of recommendations [15,93,94,98]. Therefore, future research should be directed at developing a comprehensive and up to date set of guidelines to aid reviewers in investigating clinical heterogeneity. We summarize the common recommendations that appear in the literature to offer some preliminary guidance for systematic reviewers (Table 5).

We were surprised to see that the term clinical heterogeneity was relatively commonly used and consistently defined. We took our definition from several publications with which we were previously familiar [1,3]. In some of the resources the term methodological heterogeneity was used synonymously with clinical heterogeneity, or clinical heterogeneity was considered to be one component of methodological heterogeneity. While this was infrequent in the literature, methodological aspects of heterogeneity include but go beyond clinical aspects or reasons for heterogeneity between trials. Thus, when describing reasons for heterogeneity that are related to the participants, intervention, outcomes or settings of the trial, these should be termed clinical aspects of heterogeneity. A consistency of terminology is mandatory for development of thought and investigation in this area. With terminology in place, the discussion can move to our recommendations.

When planning investigations of clinical heterogeneity in systematic reviews of controlled trials one should make such plans explicit, *a priori*, in the protocol for the review. We would suggest that protocols be published or registered in appropriate databases [118]. Next, it is reasonable and arguably beneficial, when organizing the review team, to include clinical experts or at a minimum, state a plan for consulting clinical experts during particular phases of the review (e.g., when choosing clinical covariates or during interpretation of findings). Furthermore, a set of clinical covariates should be chosen that have a clearly stated rationale for their importance (e.g., pathophysiological argument or reference to the results of a previous large trial). Review teams should think through the following categories to determine if related covariates might logically influence the treatment effect in their particular review: participant level, intervention level, outcome level, research setting, or others unique to the research question. Several resources offered conceptual mapping, idea webbing and causal modeling as possible methods for identifying important covariates and relationships between them [98,112,113]. Next, a hierarchy of clinical covariates should be formed and covariates investigated only if there is sufficient rationale and later a sufficient number of trials available. That is, covariates deemed more important than others on the basis of an explicitly stated rationale should be immediately included in such investigations, with other covariates being included when the number of trials is sufficient. A generally accepted rule of thumb is that 10 events per predictor variable (EPV) maintains bias and variability at acceptable levels. This rule derives from 2 simulation studies carried out for logistic and Cox modeling strategies [119-121] and has been adapted to meta-regression [1,114]. Therefore, it has been suggested that for each covariate there should be at least 10 trials to avoid potentially spurious findings [15]. Also, investigators should describe any plans to include additional covariates after looking at the data from included studies (e.g., forest plots). This might include an examination of summary tables or various types of plots [92,93,97,98,106], and it would be reasonable to include the clinical expert(s) at this stage to aid in the interpretation of the plotted data. Finally, how the results of any findings are going to be interpreted and used in the synthesis methods of the review needs to be explained. Most resources advise caution in interpreting these investigations, noting their exploratory nature, but when there is a clearly stated rationale, especially when derived from previous research, and sufficient trials are included, *a priori* planned investigations may improve applicability. Also, it was frequently suggested that the interpretation of the results of these investigations should consider confounds and important potential biases, the magnitude of the effect, confidence intervals and the directionality of the effect. Following these recommendations may lead to valid and reliable investigations of clinical heterogeneity and could improve their overall applicability and lead to future research that might test hypothesized subgroup effects.

A wide variety of statistical analyses are available for investigating clinical heterogeneity in systematic reviews of controlled clinical trials, and it is not within the scope of this paper to cover these in detail. Other resources cover this subject very well [15,93,95,100,114]. The sophistication of techniques is constantly growing, and an updated, precise summary of such methods is needed. Instead we will describe three available options frequently suggested by resources included in our review—subgroup analyses, meta-regression and the analogue to the analysis of variance (ANOVA)—and comment upon methods for exploring control group event rate.

Subgroup analyses involve separating trials into groups on the basis of some characteristic (e.g., intervention dose) and then performing separate meta-analyses for each group. This test provides an effect estimate within subgroups and a significance test for that estimate; it does not provide a test of variation in effect due to covariates. The greater the number of significant tests performed, the greater the likelihood of type 1 errors. There are some suggestions in the literature for how to control for this (e.g., Bonferroni adjustments [48]). To test for differences between subgroups a moderator analysis must be done. Moderator analyses include meta-regression and the analogue to the ANOVA, among other techniques (e.g., Z test [114]). Meta-regression is used to assess the impact of one or more independent variables (e.g., age or intervention dose) upon the dependent variable, the overall treatment effect [62]. Independent variables may be continuous or categorical, the latter expressed as a set of dummy variables with one omitted category. Several modeling strategies are available for performing meta-regression [100,108,122]. The results of meta-regression indicate which variables influence the summary treatment effect, how much the summary effect changes with each unit change in the variable and the p-value of this influence. It has been suggested that at least 10 trials per covariate are needed to limit spurious findings, due to the low statistical power of meta-regression, and a nonparametric test has been suggested when this tenet is not fulfilled [30] Also, one needs to consider the problems associated with ecological bias when performing meta-regressions on patient levels variables [40]. Finally, the analogue to the ANOVA examines the difference in the effect between categorical levels of some variable using identical statistical methods as a standard ANOVA [94].

The literature suggests many methods for examining the influence of the control event rate or baseline risk, which is considered an aggregate measure of known (e.g., age and disease severity) and unknown variables [15,43,93]. It has been argued that these examinations provide little import to clinical practice since the influence of any possible causative variables is aggregated and therefore the effect of individual covariates is unknown [15]. Also, the influence of the control event rate on the summary affect is affected by regression to the mean, and sophisticated statistical procedures are required to deal with this [15,43,93].

Bayesian approaches to meta-regression and hierarchical Bayes modeling, among other areas, appear to be well represented in the literature [66,71,95], as well as more general resources for Bayesian meta-analytic techniques [95,123]. These methods are developing rapidly; therefore, frequent summaries of these important techniques are required as a resource to reviewers.

Finally, we would like to note suggestions in the literature concerning the utility of aggregate patient data (APD) versus individual patient data (IPD). Several resources give general recommendations regarding use of IPD when exploring characteristics that could be considered aspects of clinical heterogeneity [15,74-76,95,97]. Some empirical evidence supports these recommendations [40,66,124,125]. When IPD is available, it should be used as a basis to investigate aspects of clinical heterogeneity at the patient level (e.g., demographic characteristics) so as to avoid ecological bias associated with summary APD. It is reasonable to use APD for trial-level covariates (e.g., intervention characteristics) that can be considered aspects of clinical heterogeneity. In addition, there may be opportunities to strategically use APD together with IPD to avoid the significant, and sometimes insurmountable, effort required to collect complete IPD [71].

Finally, in relation to the suggestions above for including clinical expertise in systematic reviews, we feel it is the responsibility of each therapeutic discipline to create a repository of variables to consider when exploring effect variation in systematic reviews. Such warehousing of clinically important covariates would serve as an important resource, allowing systematic reviewers and clinical trialists to explore nuances in treatment effect that might inform clinical decision making, and allowing for increased applicability of findings.

## Conclusions

In summary, although many recommendations are available for investigating clinical heterogeneity in systematic reviews of controlled clinical trials, there is a need to develop a comprehensive set of recommendations for how to perform valid, applicable, and appropriate investigations of clinical covariates [7,14]. This will improve the applicability and utilization of systematic reviews by policy makers, clinicians, and other decision makers and researchers who wish to build on these findings.

## Appendix A: Search strategies

### 1. OVID searches

Medline (1950 to Oct 29^th^, 2010); Cochrane Methodology Register (Oct 29^th^, 2010) ; HTA (Oct 29^th^, 2010); EMBASE (1980 to Oct 30^th^, 2010)“(((clinical adj5 heterogeneity)) and (assessment or checklist or guideline or guidelines or scale or criteria))”*Note*: A slight variation in this strategy was used for EMBASE, on the EMBASE specific search engine, for an updated search we performed from January 1^st^ 2009 to October 30^th^, 2010. This was due to a change in the available electronic resources.

### 2. CINAHL (EBSCO) (1981 up to October 30^th^, 2010)

“TX clinical N8 heterogeneity and TX ( assessment OR checklist OR guideline OR guidelines OR scale OR criteria )”

### 3. CONSORT database of methodological papers (up to Oct 30^th^, 2010)

Manual search of all citations.

### 4. Related PubMed links for (completed on October 31^st^ , 2010)

Thompson SG. Why sources of heterogeneity in meta-analysis should be investigated. BMJ. 1994;309:1351–5.

### 5. Related PubMed links for (completed on October 31^st^, 2010)

Higgins J, Thompson S, Deeks J, Altman D. Statistical heterogeneity in systematic reviews of clinical trials: a critical appraisal of guidelines and practice. Journal of Health Services & Research Policy. Jan 2002;7(1):51–61.

### 6. Related PubMed links for: (completed on October 31^st^, 2010)

**Schmid CH**, Stark PC, Berlin JA, Landais P and Lau J. Meta-regression detected associations between heterogeneous treatment effects and study-level, but not patient-level, factors.  Journal of Clinical Epidemiology. 2004;57:683–97.

## Competing interests

The authors declare that they have no competing interests.

## Authors’ contributions

JG developed conceptualized the project, searched for the literature, extracted data, and wrote the manuscript. DM, HB, JB and CB conceptualized the project and edited the manuscript. All authors read and approved the final manuscript.

## Pre-publication history

The pre-publication history for this paper can be accessed here:

http://www.biomedcentral.com/1471-2288/12/111/prepub
